# Deep learning “super resolution” versus iterative reconstruction: Phantom‐based image quality assessment

**DOI:** 10.1002/acm2.70597

**Published:** 2026-04-23

**Authors:** Stephanie M. Leon, Daniella Fabri, Colin J. Schaeffer, Edmond A. Olguin

**Affiliations:** ^1^ Department of Radiology University of Florida Gainesville Florida USA; ^2^ Department of Neurosurgery University of Florida Gainesville Florida USA; ^3^ Department of Diagnostic Radiology Oregon Health & Science University Portland Oregon USA; ^4^ Department of Radiology Beth Israel Deaconess Medical Center Boston Massachusetts USA; ^5^ Present address: Dartmouth‐Hitchcock Medical Center Department of Radiology Lebanon NH USA

**Keywords:** CT, deep learning, image quality, PIQE, reconstruction algorithms

## Abstract

**Background:**

Deep learning reconstruction (DLR) algorithms have begun replacing iterative reconstruction (IR) in CT. Besides the potential to reduce noise, the application of artificial intelligence can also allow for “super resolution,” allowing traditional CT systems to produce images with improved spatial resolution by training against higher‐resolution images. This study aims to characterize the image quality produced by a DLR “super resolution” algorithm.

**Purpose:**

This study compares Canon's Precise IQ Engine (PIQE) Cardiac, a DLR algorithm, with the AIDR Enhanced 3D iterative reconstruction (IR) algorithm using quantitative phantom‐based image quality measurements.

**Materials and methods:**

Scans of the American College of Radiology (ACR) Gammex 464 image quality phantom with and without a torso‐sized elliptical ring were acquired using CTDI_vol_ of 2.6, 15.7, and 32.9 mGy. Each experimental condition was scanned 20 times. Reconstructions used AIDR with a cardiac kernel and PIQE Cardiac with both 512 × 512 and 1024 × 1024 matrices. The task‐based modulation transfer function (MTF_task_) was calculated for Teflon, acrylic, polyethylene, and air inserts. Contrast‐to‐noise ratio (CNR) and low‐contrast object specific CNR (CNR_LO_) were calculated for the 25‐mm and 6‐mm low‐contrast disks in module 2 of the phantom. NPS and noise magnitude were calculated from the module's background.

**Results:**

Both versions of PIQE showed improvement in MTF_task_ over AIDR for most frequencies, with PIQE 1024 outperforming PIQE 512. Larger differences were noted at higher contrast levels and with the smaller phantom. NPS curves for PIQE 512 and PIQE 1024 were nearly identical at all doses. AIDR showed a noise magnitude at least 44% higher than PIQE for all scenarios. The NPS shapes differed between AIDR and PIQE, producing noticeably different noise textures that were dose‐ and phantom size‐dependent. CNR_LO_ values were similar between PIQE 512 and PIQE 1024 and were higher than those of AIDR for most scenarios; however, AIDR outperformed PIQE for the 6‐mm objects at the medium and high doses. Use of PIQE on the 25‐mm disk permits dose reductions in the small phantom without loss of CNR_LO_.

**Conclusion:**

PIQE provides substantial image quality improvements over AIDR in phantom‐based quantitative measurements. The potential for dose reduction is also noted. PIQE 1024 can be used to improve the spatial resolution over PIQE 512, with no apparent trade‐offs in image quality.

## 1 INTRODUCTION

CT image reconstruction has largely transitioned from filtered back‐projection (FBP) to iterative reconstruction (IR), which provides better suppression of noise in the image.^1^, ^2^, ^3^ Canon Medical Systems (formerly Toshiba) released its Adaptive Iterative Dose Reduction (AIDR) IR algorithm in 2010, which was followed quickly by the upgraded AIDR 3D version in 2012.^4^ Now, reconstruction technology is beginning to transition away from IR algorithms, this time to artificial intelligence (A.I.)‐based reconstruction.

Precise IQ Engine (PIQE) is the newest reconstruction algorithm released by Canon, and it uses deep learning reconstruction (DLR) to improve spatial resolution and differentiate signal from noise.^5^ The version tested in this work allows the selection of either a standard 512 × 512 image matrix (PIQE 512) or a high‐resolution 1024 × 1024 image matrix (PIQE 1024). PIQE 512 has been compared to Canon's older AI‐based reconstruction, Advanced Intelligent Clear‐IQ Engine (AiCE), using the task‐based transfer function, noise power spectrum (NPS), and a detectability index; results showed improvements in all image quality metrics.^6^ Studies looking at the application of other DLR algorithms to CT have found that DLR can improve image quality and permit dose reduction in a number of different organ systems, as summarized in the review paper by Koetzier et al.^7^ Szczykutowicz et al. used the American College of Radiology (ACR) phantom to compare another vendor's DLR algorithm to filtered back‐ projection and IR using three image quality metrics: contrast‐to‐noise ratio (CNR), modulation transfer function (MTF), and NPS; they found improvements in CNR and total noise magnitude, but no change in spatial resolution.^8^ Barca et al. looked at NPS, target‐transfer function, and CT number accuracy for a different DLR algorithm, finding reductions in noise and preservation of CT number and spatial resolution; spatial solution was contrast‐ and dose‐dependent for both the DLR algorithm and the IR algorithm to which it was compared.^9^ Yet another vendor's DLR algorithm was compared to IR with mixed results on image quality metrics: reduced noise and increased detectability were associated with decreased spatial resolution, and vice versa.^10^

PIQE is an evolution of DLR algorithms to include an emphasis on “super resolution^5^”, which trains on a CT system that can produce images acquired with both conventional spatial resolution (512 × 512 matrix with 0.5 mm detectors) and high spatial resolution (1024 × 1024 matrix with 0.25 mm detectors).^11^ An evaluation of the technology would be interesting from both a clinical and scientific perspective. PIQE 1024 has not been characterized in the literature with respect to analytical image quality parameters, and PIQE 512 has not been characterized with torso‐sized phantoms. Larger phantoms (and patients) can cause photon starvation and beam hardening artifacts, which may affect the operation of reconstruction and image processing algorithms. The impact of size is a clinically‐relevant factor that is not often considered in evaluations of image quality, and clinical studies looking at the bariatric population are lacking.^7^ Low‐contrast performance has likewise not been evaluated with analytical measurements. These characterizations are useful to provide a quantitative comparison of algorithms both within and across vendors. Thus, this work will characterize the performance of PIQE as a function of matrix size, phantom size, and three image quality metrics (spatial resolution, noise, and low‐contrast resolution).

## 2 MATERIALS AND METHODS

Scanning was performed on a Canon Aquilion ONE / PRISM Edition CT scanner (Canon Medical Systems Corp, Otawara, Japan), which has a 16‐cm longitudinal field‐of‐view and permits either helical or volumetric scanning. The scanner uses a statistical IR algorithm, AIDR 3D Enhanced (referred to simply as “AIDR” for the rest of this document), which permits the selection of a number of different kernels. For this study, kernel FC‐03 was used, which is the default selection for cardiac scans recommended by the manufacturer. The AIDR strength was set to “Standard.”

All scans were performed using the ACR 464 Phantom (Sun Nuclear, Melbourne, FL), which is the standard phantom used for image quality testing required by the ACR. This phantom was selected due to its ubiquity and high level of familiarity among physicists in the United States. The phantom contains four modules, but only Modules 1 and 2 were used in this work. Module 1 contains a background of solid water with circular test objects composed of Teflon (955 HU), acrylic (120 HU), polyethylene (‐95 HU), and air (‐1000 HU). Module 2 contains a solid water background with 6‐HU low‐contrast circular test objects; there is a large rod with a 25‐mm diameter as well as smaller rods ranging from 2–6 mm.^12^

The ACR phantom is common, but it has only a 20‐cm diameter, so it is not very representative of the attenuation from an adult torso. Because the primary indication for PIQE is currently cardiac procedures, all scans were also repeated with the ACR phantom placed within a solid‐water ellipse measuring 40 cm by 30 cm. The ellipse is part of Sun Nuclear's Multi‐Energy CT Phantom, although none of the multi‐energy inserts were used in this study. The six holes in the ellipse intended for those inserts were filled with solid water equivalent 3D‐printed inserts created in‐house (Figure 1).

**FIGURE 1:**
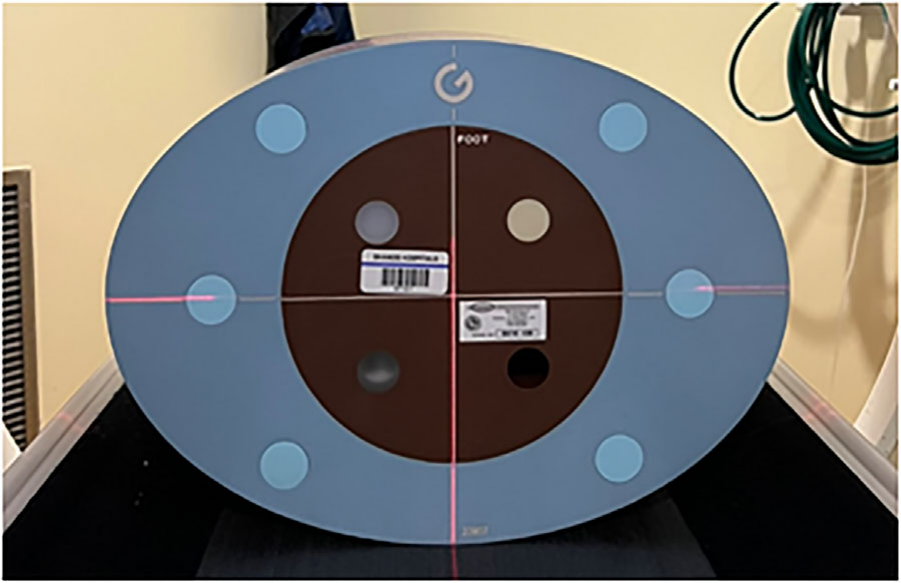
The ACR 464 phantom placed within the solid‐water ellipse. The lighter blue circles are the 3D‐printed solid water equivalent inserts.

For simplicity, the ACR phantom alone will be referred to as a “small phantom” and the combination of the ACR phantom plus ellipse will be referred to as a “large phantom.”

To cover a range of doses commonly used for clinical scans, images were acquired at 2.6, 15.7, and 32.9 mGy (as reported in the 32‐cm phantom), which used acquisition settings of 50, 300, and 600 mAs, respectively. All scans used volumetric scanning with a tube potential of 120 kV. The small bowtie filter was used for the small phantom, and the large bowtie filter was used for the large phantom. The focal spot was selected automatically by the scanner on the basis of the tube current used; the small focal spot was used for 50 and 300 mAs, and the large focal spot was used at 600 mAs. Focal spot selection is not under the control of the user. Each combination of imaging parameters was acquired 20 times.

Raw data was saved onto an external hard drive, which was sent to Canon for reconstruction with AIDR, PIQE 512, and PIQE 1024 (which was not commercially available at the time of the experiment). The display field‐of‐view (DFOV) used for the small phantom was 210.9 mm; for the large phantom, reconstructions were performed both with a DFOV of 419.9 mm (which showed the entire phantom) and 210.9 mm (which showed only the central portion). The slice thickness for all reconstructions was 0.5 mm.

For both phantom sizes, image quality was assessed by measurement of the task‐based MTF_task_,^13^ the noise power spectrum (NPS), and low‐contrast resolution (CNR). An additional metric used was the low‐contrast object specific CNR (CNR_LO_), which is dependent on noise frequency and has been found to predict visualization of low‐contrast objects better than the CNR (Equation 1).^14^ It is defined as:

(1) $$CNR_{LO}\left(\bar{u}\right)=\frac{S_{disk}-S_{bg}}{\sqrt{NPS\left(\bar{u}\right)}}\;$$

where S is the signal, σ is the standard deviation, $\bar{u}$ is the spatial frequency corresponding to the diameter of the disk, and $NPS(\bar{u})$ is the NPS at that frequency. Python codes created in‐house were used for all measurements.

MTF_task_ was measured on the 210.9 mm reconstruction of Module 1 of the ACR phantom, using a circular‐edge technique to measure the edge‐spread function (ESF) for each scan/reconstruction combination across each of the four test objects.^15^ Pixel values in each slice were sampled radially to generate profiles with 8100 data points for each insert. Then, 30 central slices from a single scan were averaged together. Lastly, the curves from all 20 scans were averaged. The limiting spatial frequency was reported as the value for which the MTF_task_ reduces to 10%.

The NPS was measured on Module 2 of the ACR phantom. NPS was calculated in Module 2 instead of Module 3 (the uniformity section) to give a more accurate estimate of noise for the CNR_LO_ calculation described in the next section. The NPS was calculated from 20 repeated scans of module 2 of the ACR phantom using the central 40 slices from each scan. The subtraction method was used to subtract non‐stochastic features, leaving an image of pure noise.^16^ In each subtracted slice, 32 ROIs (64 × 64 for the 512 recons and 128 × 128 for the 1024 recons) were placed at uniform angular intervals and at the radius of the low contrast objects. A 2D NPS was calculated from each ROI and was averaged over all ROIs and slices to create a final 2D NPS. The 1D NPS was then extracted from the 2D NPS by rebinning each pixel according to its radial frequency. The areas under the NPS curves were calculated and compared for significant differences using a two‐tailed *t*‐test with a significance level of 0.05. The NPS was then divided by its magnitude to display the normalized noise power spectrum (NNPS).

Low contrast resolution was measured using both the CNR and CNR_LO_ metrics; CNR_LO_ considers the noise frequencies present in the image, whereas the traditional CNR has no frequency‐dependence.^14^ The 25‐mm and 6‐mm low contrast disks in ACR phantom module 2 were used for these measurements. The reported values are the means and standard errors of measurements from 800 individual images from each dose/reconstruction combination. Differences in the measurements were tested for significance using a two‐tailed *t*‐test with *p *< 0.05 considered significant.

## 3 RESULTS

### 3.1 Spatial resolution

Results for MTF_task_ for each insert material are shown in Figures 2, 3, 4, and are discussed separately for the small and large phantoms, below. Limiting spatial resolutions are summarized in Table 1.

**FIGURE 2:**
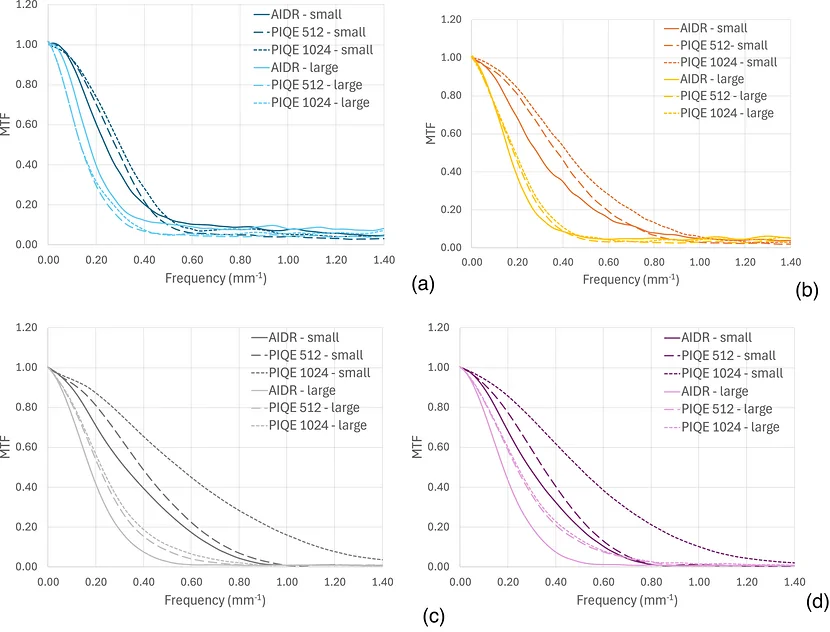
MTF_task_ curves for the small and large phantoms with the 2.6 mGy dose for (a) polyethylene, (b) acrylic, (c) Teflon, and (d) air. All reconstructions were created with the same (210.9 mm) display field of view.

**FIGURE 3:**
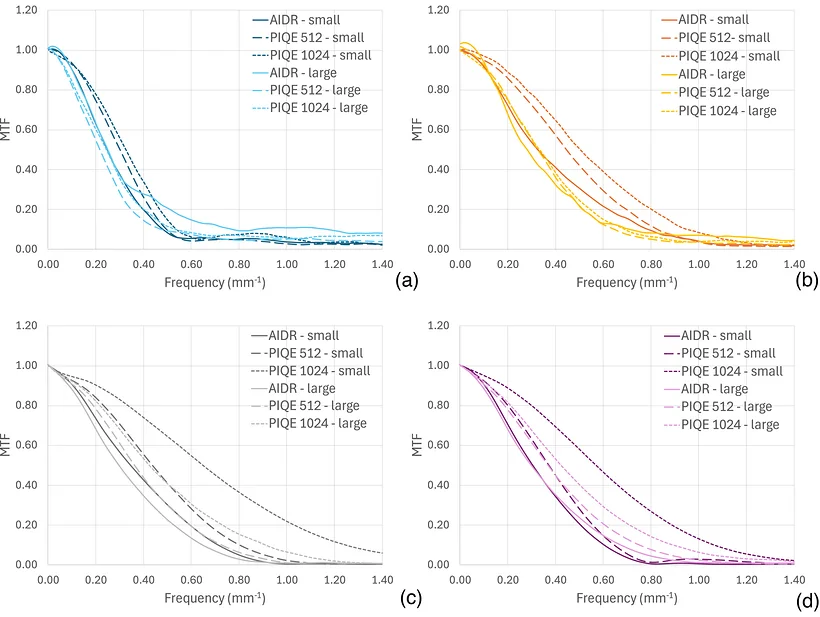
MTF_task_ curves for the small and large phantoms with the 15.7 mGy dose for (a) polyethylene, (b) acrylic, (c) Teflon, and (d) air. All reconstructions were created with the same (210.9 mm) display field of view.

**FIGURE 4:**
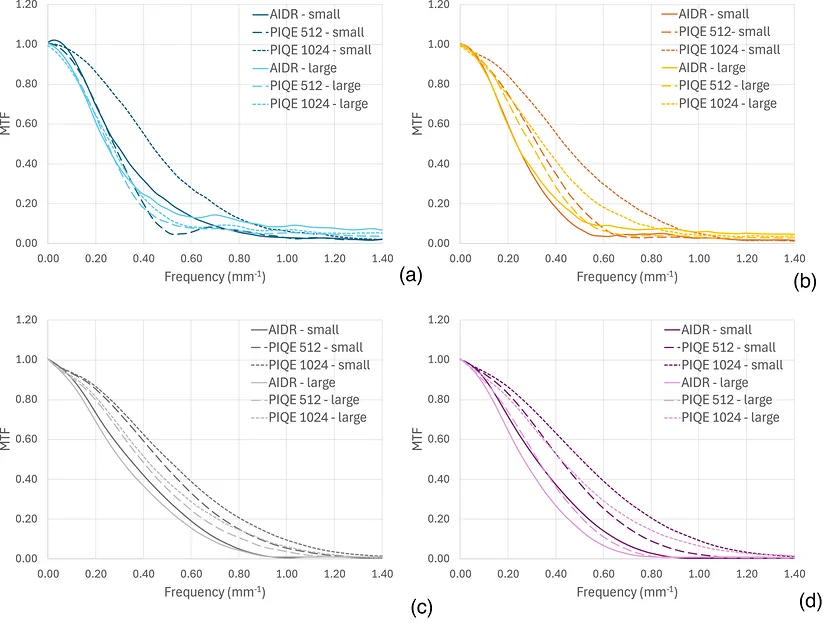
MTF_task_ curves for the small and large phantoms with the 32.9 mGy dose for (a) polyethylene, (b) acrylic, (c) Teflon, and (d) air. All reconstructions were created with the same (210.9 mm) display field of view.

**TABLE 1:** Limiting frequencies (in mm−1) where MTFtask equals 10% for each insert, each dose level, and each reconstruction algorithm using the each phantom size. The reconstructed field of view was the same (210.9 mm) for both phantom sizes.

	AIDR 3D enhanced			PIQE 512			PIQE 1024		
Insert	Low dose	Medium dose	High dose	Low dose	Medium dose	High dose	Low dose	Medium dose	High dose
Small phantom									
Air	0.606 ± 0.005	0.606 ± 0.003	0.654 ± 0.007	0.637 ± 0.005	0.645 ± 0.002	0.786 ± 0.011	1.003 ± 0.008	1.064 ± 0.002	0.986 ± 0.006
Polyethylene	0.638 ± 0.035	0.483 ± 0.035	0.668 ± 0.026	0.510 ± 0.040	0.496 ± 0.019	0.451 ± 0.023	0.554 ± 0.030	0.752 ± 0.023	0.870 ± 0.023
Acrylic	0.762 ± 0.031	0.806 ± 0.018	0.476 ± 0.041	0.731 ± 0.022	0.835 ± 0.013	0.571 ± 0.041	0.870 ± 0.023	0.993 ± 0.016	0.857 ± 0.015
Teflon	0.696 ± 0.006	0.714 ± 0.003	0.712 ± 0.005	0.745 ± 0.010	0.804 ± 0.001	0.886 ± 0.004	1.129 ± 0.010	1.244 ± 0.002	0.984 ± 0.008
Large phantom									
Air	0.372 ± 0.008	0.672 ± 0.008	0.553 ± 0.007	0.545 ± 0.004	0.749 ± 0.004	0.613 ± 0.003	0.561 ± 0.008	0.892 ± 0.03	0.892 ± 0.020
Polyethylene	0.477 ± 0.049	0.958 ± 0.050	0.843 ± 0.030	0.341 ± 0.050	0.468 ± 0.048	0.512 ± 0.046	0.353 ± 0.038	0.529 ± 0.042	0.741 ± 0.039
Acrylic	0.355 ± 0.059	0.709 ± 0.054	0.555 ± 0.039	0.390 ± 0.024	0.635 ± 0.034	0.529 ± 0.032	0.415 ± 0.030	0.705 ± 0.039	0.748 ± 0.027
Teflon	0.377 ± 0.012	0.650 ± 0.008	0.678 ± 0.010	0.466 ± 0.010	0.727 ± 0.005	0.816 ± 0.004	0.529 ± 0.005	0.908 ± 0.006	0.897 ± 0.013

### 3.2 Small phantom

For the relatively low‐contrast polyethylene insert, PIQE has little benefit at low and medium doses; at high doses, only PIQE 1024 shows a benefit. For all other inserts, the MTF_task_ curves progress as follows: AIDR demonstrates the worst performance, PIQE 512 shows some improvement, and PIQE 1024 shows the greatest improvement.

For both PIQE 512 and PIQE 1024, MTF_task_ improves moving from the low to the medium dose. However, the MTF_task_ measurements with the high dose demonstrate a material dependence. For PIQE 512, performance decreases at high dose with the low‐contrast polyethylene and acrylic inserts, but improves for the high‐contrast Teflon and air inserts.

Limiting spatial resolutions (Table 1) generally support the trends noted for the curves as a whole.

### 3.3 Large phantom

At the low dose, the MTF_task_ curves for the large phantom show worse performance than for the small phantom for all materials and all reconstruction algorithms. The performance of PIQE with the polyethylene insert is actually slightly worse than AIDR at higher frequencies for all dose levels. PIQE provides no benefit over AIDR for the acrylic inserts at the low and medium doses but does begin to improve performance with the higher‐contrast Teflon and air. PIQE 1024 outperforms PIQE 512 with these materials, but not to the extent that it did with the small phantom.

The behavior of the algorithms at the high dose is again material‐dependent. All algorithms perform similarly for polyethylene at frequencies below 0.4 mm^−1^, after which they diverge at the higher frequencies; for acrylic, PIQE 512 and 1024 show some improvement at lower frequencies but become similar to AIDR at frequencies above 0.8 mm^−1^. For Teflon and air, both PIQE versions provide a benefit over AIDR, but the benefit of PIQE 1024 is more pronounced for air.

Similarly to the small phantom, MTF_task_ improves when moving from the low to the medium dose for both PIQE 512 and PIQE 1024. The MTF_task_ measurements with the high dose once again demonstrate a material dependence. For PIQE 512, performance improves at high dose for polyethylene and Teflon but decreases for acrylic and air. For PIQE 1024, only polyethylene improves at high dose, with performance decreasing for the other inserts; this is the same trend as was observed on the small phantom and is likely related to the focal spot size.

### 3.4 NPS

There are stark differences in the NNPS when comparing AIDR to PIQE (Figure 5; Table 2).

**FIGURE 5:**
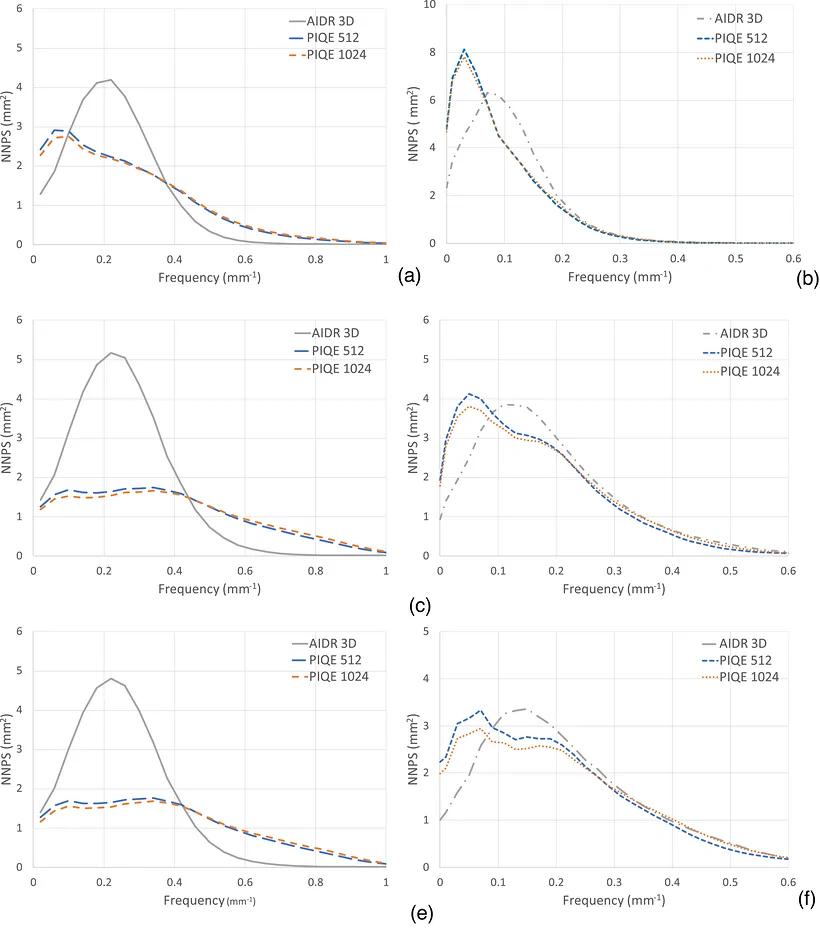
NPS data for AIDR, PIQE 512, and PIQE 1024 showing (a) small phantom at 2.6 mGy, (b) large phantom at 2.6 mGy, (c) small phantom at 15.7 mGy, (d) large phantom at 15.7 mGy, (e) small phantom at 32.9 mGy, and (f) large phantom at 32.9 mGy.

**TABLE 2:** Peak frequencies (in mm−1) of the NPS for each phantom size, dose level, and reconstruction algorithm.

	AIDR 3D enhanced			PIQE 512			PIQE 1024		
Phantom	Low dose	Medium dose	High dose	Low dose	Medium dose	High dose	Low dose	Medium dose	High dose
Small	0.18	0.18	0.18	0.06	0.34	0.34	0.10	0.34	0.34
Large	0.07	0.11	0.15	0.03	0.05	0.07	0.03	0.05	0.07

With the small phantom, both PIQE algorithms produce a much flatter curve than AIDR. The square root of the area under the curve (AUC) quantifies the differences in total noise magnitude, and is shown in Figure 6. Due to the very high number of measurements (12 800 per dose/reconstruction combination), all differences were statistically significant at *p *< 0.0001, although the clinical importance of the differences between PIQE 512 and PIQE 1024 is likely negligible. The difference in appearance between AIDR and PIQE can be seen in Figure 7a and 7b; PIQE has a finer noise texture and less noise overall.

**FIGURE 6:**
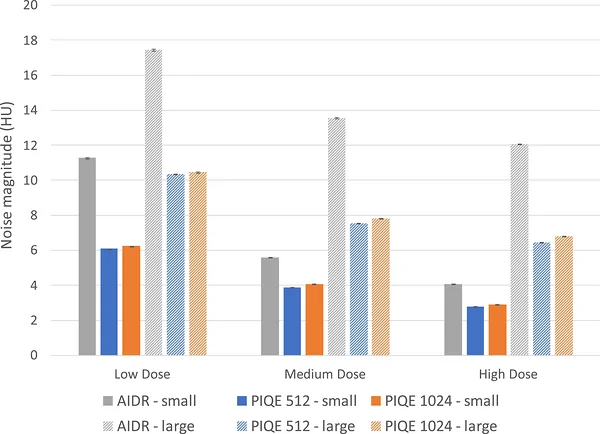
Area under the curve and SE for each dose/reconstruction/phantom combination.

**FIGURE 7:**
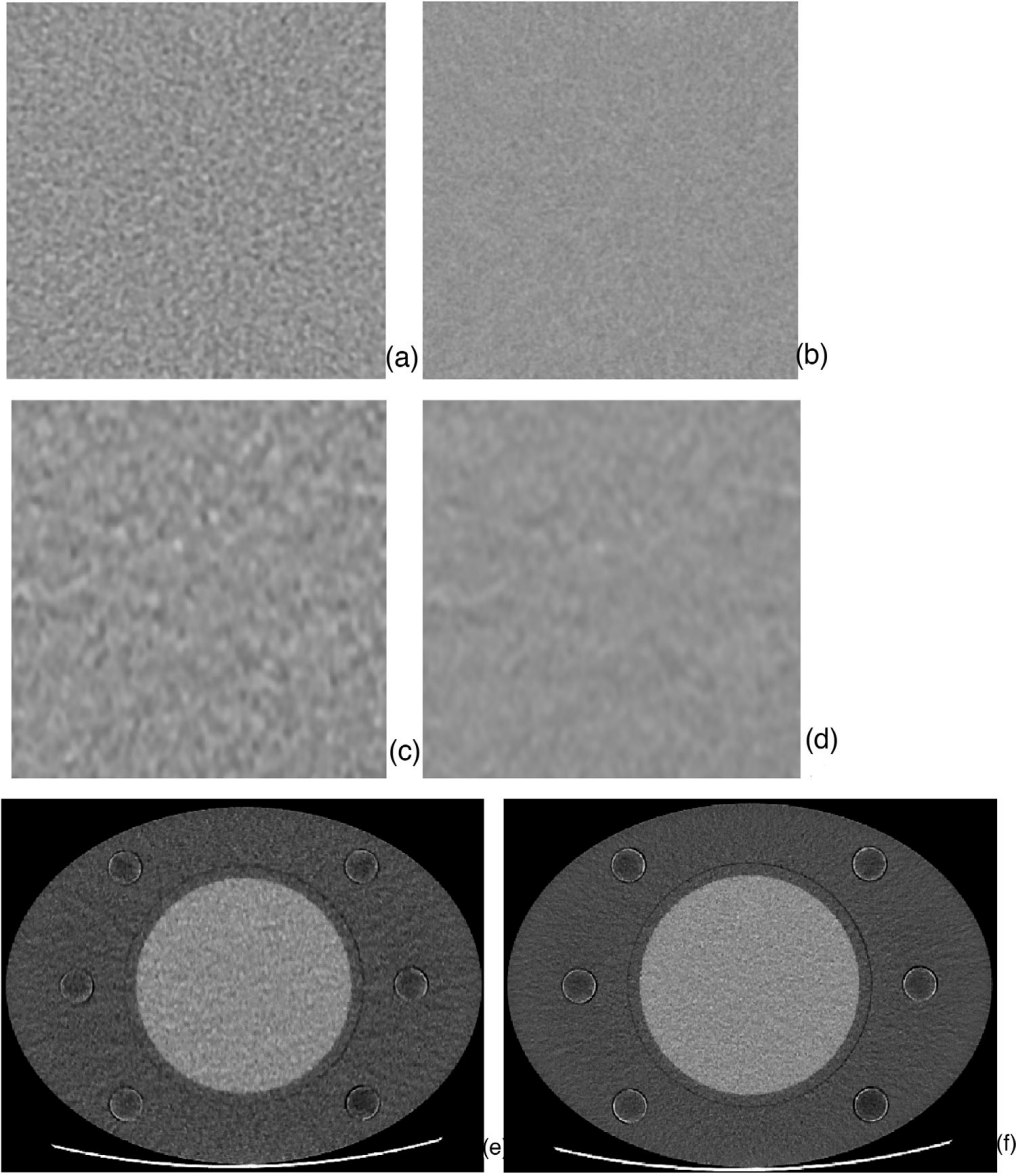
Noise background for (a) small phantom with AIDR, (b) small phantom with PIQE 512, (c) large phantom with AIDR, (d) large phantom with PIQE 512. Images A–D were acquired at the low dose and have the same window and level settings. The large phantom is also shown using PIQE 512 with (e) the 2.6 mGy dose, and (f) the 15.7 mGy dose.

With the large phantom, the peak frequency is lower for PIQE than for AIDR at all doses (Table 2), which implies that the images would have a coarser noise texture; however, the overall noise level is so much lower for PIQE than for AIDR that this difference is somewhat difficult to visualize (Figure 7c and 7d). The PIQE spectra become progressively flatter as the dose increases. This change in noise texture can be visualized to scale with the phantom in Figure 7e and 7f.

### 3.5 Contrast performance

Contrast performance is quantified both by the traditional CNR and CNR_LO_ (Figure 8). CNR is included mainly because it is such a ubiquitous metric; however, CNR_LO_ better reflects the visual detectability of low‐contrast objects when comparing images with differing noise spectra.^14^, ^15^

**FIGURE 8:**
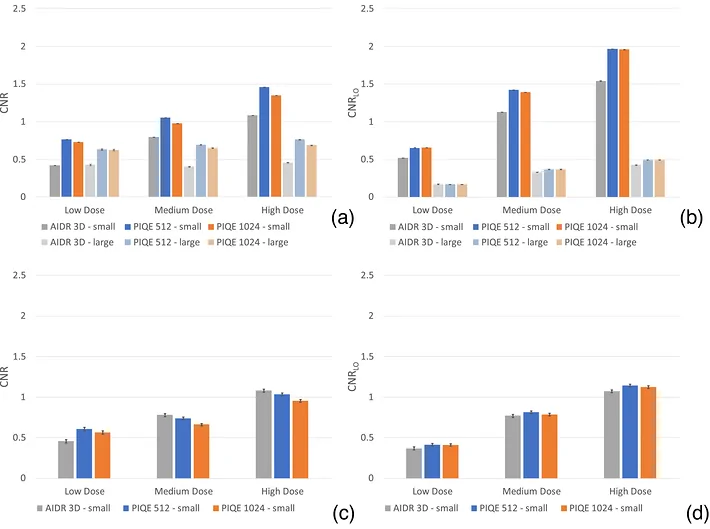
For each dose/reconstruction combination, (a) CNR for the 25‐mm disk, (b) CNR_LO_ for the 25‐mm disk, (c) CNR for the 6‐mm disk, and (d) CNR_LO_ for the 6‐mm disk.

For the larger 25‐mm low‐contrast object on the small phantom, the CNR data show that PIQE performs better than AIDR (*p *< 0.0001), with PIQE 512 outperforming PIQE 1024 (*p *< 0.0001). CNR_LO_ likewise indicates that PIQE is superior to AIDR, but in this case, PIQE 512 and PIQE 1024 show much less difference between them. There is a significant difference at the medium dose (*p *< 0.0001), but not at the low or high doses (*p *= 0.52 and 0.17, respectively). None of the differences between PIQE 512 and PIQE 1024 are likely to be noticed clinically (Figure 9). Of particular interest is the fact that, in the small phantom, CNR_LO_ measurements with the 25‐mm object at 15.7 mGy with PIQE were non‐inferior to those at 32.9 mGy with AIDR (*p *= 0.40), which suggests a potential for dose savings with PIQE.

**FIGURE 9:**
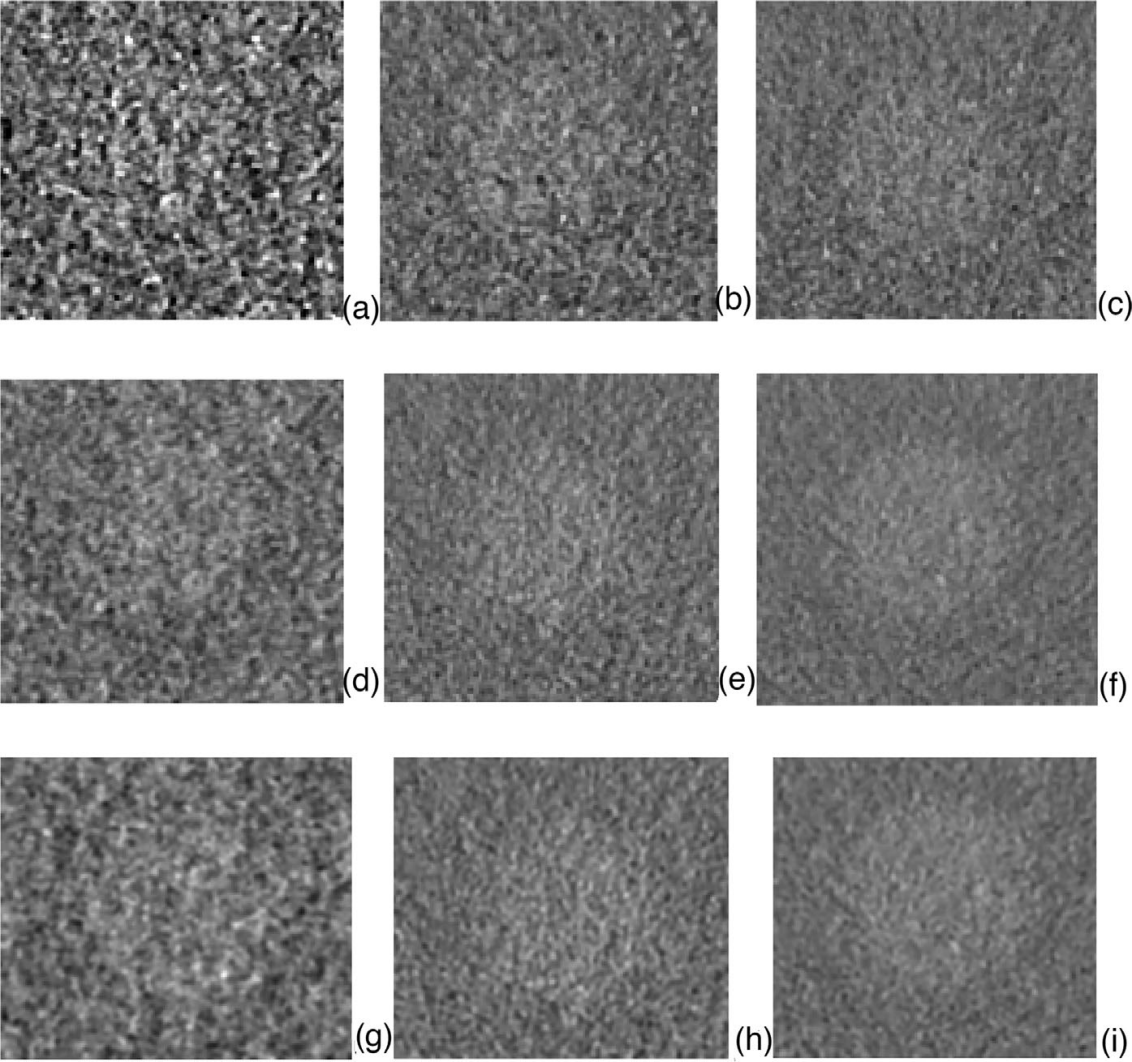
The 25‐mm low‐contrast test object is shown for (a) low dose AIDR, (b) medium dose AIDR, (c) high dose AIDR, (d) low dose PIQE 512, (e) medium dose PIQE 512, (f) high dose PIQE 512, (g) low dose PIQE 1024, (h) medium dose PIQE 1024, and (i) high dose PIQE 1024.

For the 25‐mm disk on the large phantom, the values for both CNR and CNR_LO_ are lower than those for the small phantom, as expected due to the higher noise levels. The drop from the small to the large phantom is more pronounced for CNR_LO_, and it is constant across dose levels: AIDR drops by ∼70% and PIQE (both 512 and 1024) by 75%.

The data trends for the large phantom for CNR appear very similar to those for the small phantom; the only difference is that PIQE 512 and PIQE 1024 are not significantly different at the low dose (*p *= 0.54). For CNR_LO_, the differences between AIDR and PIQE at the medium and high doses are significant at *p *< 0.0001, but there is no significant difference at the low dose (*p *= 0.34). The differences between PIQE 512 and PIQE 1024 are not significantly different at any dose level (*p* = 0.60, 0.50, and 0.65 for the low, medium, and high doses, respectively).

For the smaller 6‐mm disk, the CNR trends using the small phantom are quite different than they were for the 25‐mm phantom: PIQE 512 is best at the low dose, but AIDR outperforms PIQE at the medium and high doses (all *p *< 0.0001). At the low and medium doses, AIDR performs equivalently to PIQE on the CNR_LO_ metric, with all *p*‐values above 0.05. At the high dose, however, PIQE does show an improvement in CNR_LO_ over AIDR (*p *= 0.0026 for PIQE 512 and *p *= 0.029 for PIQE 1024). There is little difference between PIQE 512 and PIQE 1024, with *p *= 0.85 at the low dose, *p *= 0.22 at the medium dose, and *p *= 0.38 for the high dose.

The 6‐mm disk was not visible with the large phantom on any of the images, so CNR and CNR_LO_ measurements were not calculated.

Using CNR_LO_, PIQE is superior to AIDR for almost all cases tested with the 25‐mm contrast object, although the improvement is less dramatic using the larger phantom size. In the small phantom, the improvement in CNR_LO_ averaged 26% for the 25‐mm test object but only 7% for the 6‐mm test object. Differences between PIQE 512 and PIQE 1024 were small as they relates to CNR_LO_.

## 4 DISCUSSION

At this time, PIQE is intended for cardiac imaging, which presents both low‐contrast and high‐contrast challenges due to the inclusion of soft tissue, bone, iodine contrast, and lung. Additionally, the objects of interest range dramatically in size. Thus, all of the image quality tests presented here have applicability to the task at hand.

When imaging the smaller phantom, PIQE shows benefits for spatial resolution when the object has high contrast with the background, regardless of dose level. Although clinical claims based on phantom studies must be made carefully, it is possible that small vessels and stents in cardiac imaging might be more clearly visible using PIQE; the phantom images shown in the top and middle rows of Figure 10 seem to support this idea. Other DLR algorithms have been shown to improve the sharpness of small cardiac structures in clinical studies.^7^, ^17^ PIQE 1024 also shows the potential of improving the spatial resolution at lower‐contrast boundaries when higher doses are utilized. This benefit largely disappears for the lowest‐contrast insert at the low and medium doses, however. Other researchers have likewise found a decline in spatial resolution for low‐contrast objects at lower doses,^3^, ^18^, ^19^ so this may be a general limitation of DLR that remains to be addressed. Possibly, the blame may lie in insufficient relevant training data or an inability to translate the training to phantom tests. For example, DLR has been shown to blur objects that are underrepresented in the training data.^17^ DLR also may depend on training data reconstructed with algorithms that struggle with low‐contrast objects.^20^, ^21^

**FIGURE 10:**
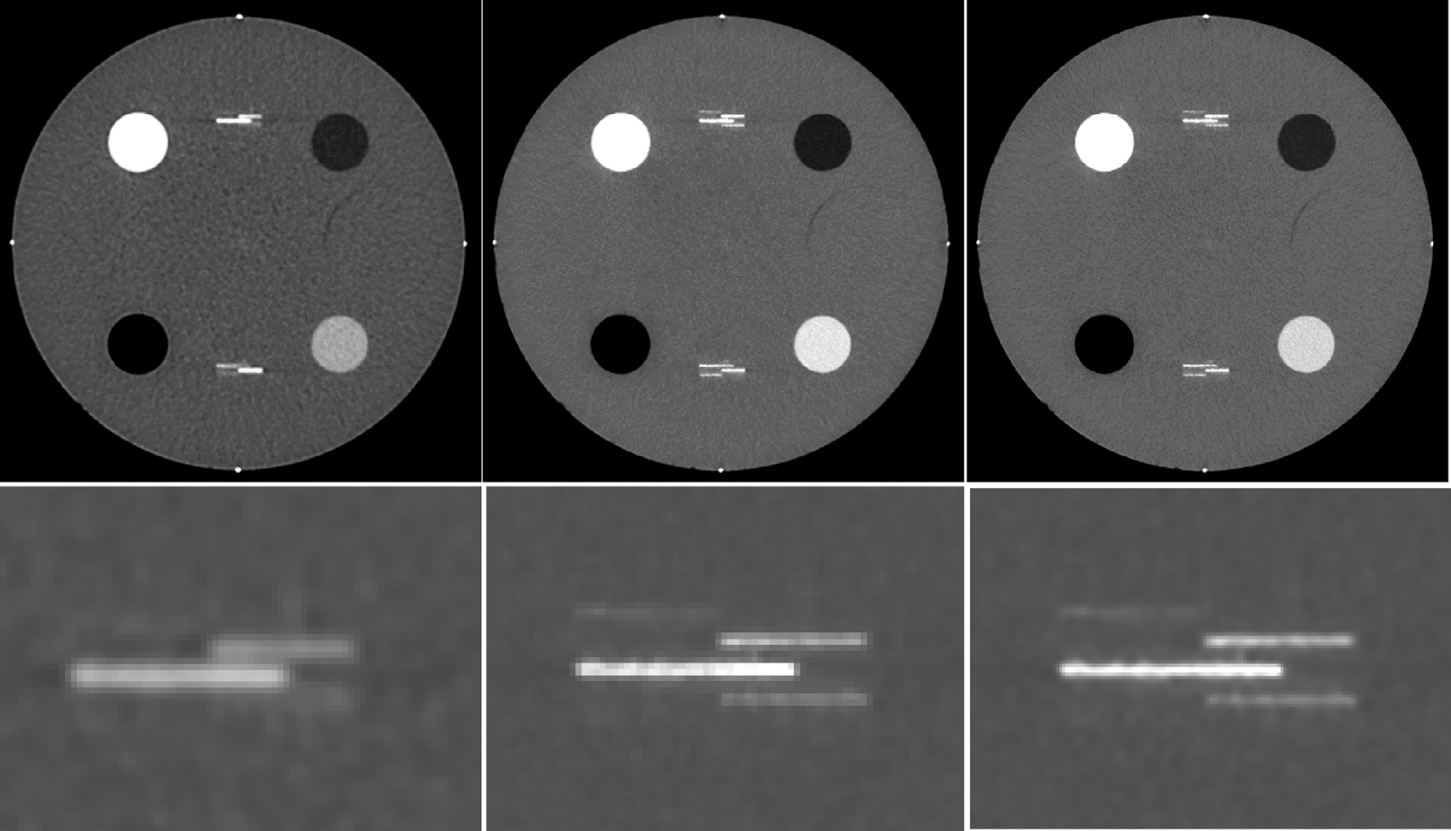
Images of the ACR phantom reconstructed with AIDR 3D (left), PIQE 512 (center), and PIQE 1024 (right). The top row shows Module 1 of the small phantom and the middle row shows a magnified view of Module 1.

The large phantom also demonstrates benefits to spatial resolution from the application of PIQE, although less dramatically than the small phantom, and likewise excluding the lowest‐contrast insert. In fact, PIQE produces an MTF_task_ for polyethylene in the large phantom that is inferior to AIDR at the higher frequencies. The complex way that these algorithms respond to dose, contrast, and object size demonstrates the importance of using MTF_task_ in order to fully characterize the system under different scenarios. An additional complicating factor that affects spatial resolution comparisons is the change in focal spot size at higher doses. The change in matrix size is likewise a complicating factor; although the Nyquist frequency with the 210 mm‐FOV would in principle be doubled from 1.2 to 2.4 mm^−1^, the MTF_task_ does not extend that far for any material, which suggests that other factors such as focal spot size, detector element size, and image processing dominate the spatial resolution.

The NPS data can be validated to a degree by comparison with the study of PIQE 512 by Greffier et al.^6^ They used only the small phantom and did not cover the same range of dose levels as this study, which limits the comparison to the small phantom at the lowest dose level, but both the shape of the NPS curve and the total noise magnitude are similar. At 3.1 mGy, they found a noise magnitude of 9.8 HU with an average frequency of 0.29 mm^−1^; at the slightly lower dose of 2.6 mGy, we found a noise magnitude of 11.2 HU and an average frequency of 0.26 mm^−1^. The NPS data is also qualitatively similar in shape to the DLR NPS curves published by Brady et al.^22^

One observation apparent in Figures 5 and 7 is that the performance of PIQE at different phantom sizes changes more dramatically than did the older AIDR algorithm and could result in more noticeable variations in noise texture among patients. When clinical studies are performed in the future, it would be valuable for them to include patients of widely varying sizes, for example, by including younger pediatric patients as well as larger adults.

Regarding contrast, the improvement in CNR_LO_ from AIDR compared to PIQE was greater for the larger test object than the smaller one. Clinically, this suggests that PIQE could provide a benefit when imaging large, low‐contrast objects. For small objects, it improves high‐contrast resolution (as evidenced by the MTF curves) but has minimal impact on low‐contrast features. This scenario describes cardiac imaging well, which contains small high‐contrast vessels and stents in a background of low‐contrast tissue.

With the large phantom, the improvement in CNR_LO_ ranged from nil at the low dose to 16% at the high dose. None of the algorithms were able to display the 6‐mm test object in the large phantom. Although the exterior dimensions of the large phantom are more similar to an adult torso than the small phantom's, it should be noted that the total attenuation of the large phantom is higher than a patient's due to the absence of lung cavities. It is unknown to what extent object shape and attenuation independently impact the AIDR and PIQE algorithms.

The finding of some CNR_LO_ measurements to be non‐inferior using PIQE at 15.7 mGy compared to AIDR at 32.9 mGy is suggestive of a potential for dose reduction; several clinical studies have suggested that reductions above 40% are possible.^23^, ^24^, ^25^, ^26^, ^27^ Using PIQE instead of AIDR in a smaller patient could preserve low‐contrast detectability at a lower dose, a finding that is particularly of interest for pediatric applications. For the larger patient, PIQE cannot compensate for a large dose reduction, but it is possible that a smaller yet still sizeable dose reduction may be feasible. Assessment of the exact level of dose reduction, as well as determination of the “best” dose for different clinically‐applicable tests of detectability, remains a topic for future work.

Put together, these phantom‐based measurements indicate that PIQE provides superior image quality over AIDR in most contexts. Additionally, there appears to be no reason (other than perhaps file sizes) not to use PIQE 1024 over PIQE 512, since it provides superior spatial resolution without increased noise or loss of low‐contrast resolution. However, there are limitations to the number of parameters that were tested; for example, only 120 kV was utilized, and only one AIDR kernel and strength were assessed.

It should be noted that all phantom studies have limitations, since no phantom can perfectly mimic patient anatomy. Whether these measured parameters will translate into clinically‐appreciable improvements is unknown; however, the perceived differences are fairly dramatic, suggesting that a noticeable clinical impact is more likely than not. Future studies should investigate the differences between AIDR, PIQE 512, and PIQE 1024 in a clinical setting, which is likely to present image quality challenges not represented by the phantom. For instance, none of the features tested in this study are applicable to small high‐contrast objects such as stents or iodinated vessels. Future applications of PIQE for noncardiac indications should also be investigated. Regardless, use of a standardized phantom allows the benchmarking and performance evaluation of technologies under controlled conditions. Thus, phantom studies remain the appropriate choice for providing quantitative comparisons among imaging systems.

## 5 CONCLUSION

Our quantitative phantom‐based findings suggest that the DLR‐based PIQE reconstruction algorithm can provide substantial image quality improvements over AIDR 3D Enhanced. In tests using phantoms of two different sizes and scanned with three different dose levels, PIQE demonstrated superior spatial resolution, lower noise, and improved contrast detectability in most scenarios. PIQE 1024 can be used to improve the spatial resolution over PIQE 512, with no apparent trade‐offs in image quality.

## AUTHOR CONTRIBUTIONS

Leon participated in the study design, data collection, data analysis, and writing the manuscript. Fabri participated in study design, data collection and processing, and manuscript revision. Schaeffer participated in study design, data analysis, and manuscript revision. Olguin participated in study design, data analysis, and manuscript revision. All authors have approved the final version of the manuscript.

## CONFLICT OF INTEREST STATEMENT

Stephanie M. Leon—Research agreement with Canon Medical Systems. Daniella Fabri, Colin J. Schaeffer, Colin J. Schaeffer—No relevant conflicts of interest to disclose

## ETHICS STATEMENT

This study did not use patient data, so no IRB approval was required.

## Data Availability

Data is available upon request
